# Association of Opioid Use Disorder With 2016 Presidential Voting Patterns: Cross-sectional Study in New York State at Census Tract Level

**DOI:** 10.2196/23426

**Published:** 2021-04-21

**Authors:** Anthony Xiang, Wei Hou, Sina Rashidian, Richard N Rosenthal, Kayley Abell-Hart, Xia Zhao, Fusheng Wang

**Affiliations:** 1 Stony Brook University Stony Brook, NY United States; 2 Renaissance School of Medicine at Stony Brook University Stony Brook, NY United States

**Keywords:** opioid use disorder, opioid poisoning, racial and ethnic disparities, geographic variance, sociodemographic factors, presidential election

## Abstract

**Background:**

Opioid overdose-related deaths have increased dramatically in recent years. Combating the opioid epidemic requires better understanding of the epidemiology of opioid poisoning (OP) and opioid use disorder (OUD).

**Objective:**

We aimed to discover geospatial patterns in nonmedical opioid use and its correlations with demographic features related to despair and economic hardship, most notably the US presidential voting patterns in 2016 at census tract level in New York State.

**Methods:**

This cross-sectional analysis used data from New York Statewide Planning and Research Cooperative System claims data and the presidential voting results of 2016 in New York State from the Harvard Election Data Archive. We included 63,958 patients who had at least one OUD diagnosis between 2010 and 2016 and 36,004 patients with at least one OP diagnosis between 2012 and 2016. Geospatial mappings were created to compare areas of New York in OUD rates and presidential voting patterns. A multiple regression model examines the extent that certain factors explain OUD rate variation.

**Results:**

Several areas shared similar patterns of OUD rates and Republican vote: census tracts in western New York, central New York, and Suffolk County. The correlation between OUD rates and the Republican vote was .38 (*P*<.001). The regression model with census tract level of demographic and socioeconomic factors explains 30% of the variance in OUD rates, with disability and Republican vote as the most significant predictors.

**Conclusions:**

At the census tract level, OUD rates were positively correlated with Republican support in the 2016 presidential election, disability, unemployment, and unmarried status. Socioeconomic and demographic despair-related features explain a large portion of the association between the Republican vote and OUD. Together, these findings underscore the importance of socioeconomic interventions in combating the opioid epidemic.

## Introduction

The United States is experiencing an epidemic of nonmedical opioid use involving both prescribed pain relievers and illegal drugs such as heroin and fentanyl. In 2017, the US Department of Health and Human Services declared the opioid crisis a public health emergency [1]. Geographic variation is a crucial factor in studying patterns in opioid deaths. Previous studies have shown that certain state- or county-level characteristics such as rurality, poverty, educational attainment, health care access, and racial demographics are associated with higher opioid use [2-4]. Opioid use disorder (OUD) is defined as a problematic pattern of opioid use leading to problems or distress with at least 2 use-related symptoms over a 12-month period, including impaired control (eg, craving, desire to cut down, taking more than intended), social impairment (eg, social or interpersonal problems, reduction in important activities), risky use (use in hazardous situations, continued use that worsens a physical or mental problem), or noniatrogenic tolerance and/or withdrawal [5]. The public face of the opioid epidemic has been represented by the increasing prevalence of opioid-related drug overdoses and resulting fatalities, typically due to respiratory depression [1]. Whether fatal or not, these diagnoses of opioid overdoses are commonly represented in health databases as opioid poisoning (OP) events [6].

An earlier study observed similarities between geographic variation of opioid use and Republican voters at the county level [7]. Rather than being directly causal, this association is likely driven by external factors shared by both opioid users and voters for the Republican candidate in the 2016 election. Understanding the nature of this relationship helps to place the opioid epidemic in its larger sociopolitical context and further illuminates the importance of addressing socioeconomic factors in order to fight the opioid epidemic. Prior analysis suggested higher rates of county-level public health measures such as physically unhealthy days, mentally unhealthy days, age-adjusted mortality rate, teen births, diabetes, and obesity were associated with shifting to the Republican presidential candidate in the 2016 election [8]. Because OUD and OP are associated with both physical and mental distress, which can be proxied by the above measures, we explored the relationship of OUD and OP to demographic and other variables including voting for the Republican candidate [9].

While the prevalence of OUD is greater than that of OP, there is certainly overlap in OP and OUD populations, since OUD is a major risk factor for opioid overdose; however, significant OP also occurs in subpopulations not identified as high risk (high risk being those with chronic opioid use, nonmedical opioid use, OUD) [6,10]. As such, we chose to investigate these related but nonidentical populations at the census tract (CT) level in respect to voting patterns in the 2016 presidential election.

Our aim is to better understand the interconnected relationship between opioid use, Republican voting, and other demographic factors in New York State. Our analysis is at the CT level, which provides a much higher resolution than previous studies. Census tracts generally contain between 2500 to 8000 people [11], far fewer than the 100,000-inhabitant average at the county level. This fine-grained analysis makes our spatial correlations much more powerful, better revealing how different factors contribute to OUD and OP in communities across New York State.

## Methods

This study was approved by the Stony Brook University institutional review board and the Office of Quality and Patient Safety, Department of Health of New York State. Informed consent was not needed as the study had no contact with participants and the data were obtained from a New York State administrative database. The primary research question and analysis plan were not preregistered on a publicly available platform, and thus the results should be considered exploratory.

### Data Collection

The presidential voting results of 2016 were obtained from the Harvard Election Data Archive [12]. These data provided the number of votes for each candidate at an election precinct level, a geographic region generally smaller than the CT level. Several counties (eg, Wyoming County) had incomplete or incoherent data, so those counties were contacted directly to provide election data. The dataset was joined to a geospatial, precinct-level shapefile in ArcGIS Desktop 10.7.1 (Esri). The precinct-level voting data was extrapolated to the larger CT level by area-based estimation (Multimedia Appendix 1). The CT voting counts were a linear combination of the precinct-level voting counts and precinct area percentage within that CT (CT components add up to 1). The number of votes for each candidate was then normalized by US Census population estimates of each CT.

The demographic data were taken from the American Community Survey (ACS) by the US Census Bureau. CT level education, age, marriage, unemployment, income, population, race, gender, disability, and health care data (Medicare and Medicaid eligibility) were provided in the 2012-2016 ACS 5-year estimates [13]. These data were mapped to a CT shapefile. Urban-ness is taken from the 2010 Census Summary File and is calculated as the number of households living in an urban area divided by the total number of households in the CT [14].

The opioid-related patient information was extracted from the Statewide Planning and Research Cooperative System (SPARCS) database, a central administrative repository for health event claims data for New York State patients [6]. We extracted patients based on International Classification of Diseases (ICD) codes (primary and secondary diagnosis codes, ICD-9 from January 1, 2012, to September 30, 2015, and ICD-10 from October 1, 2015, to December 31, 2016). Two cohorts of patients were extracted; first, patients diagnosed with OP (Multimedia Appendix 2) between 2012 and 2016, and second, patients diagnosed with OUD (Multimedia Appendix 3) between 2010 and 2016. For converting home addresses to geolocations (latitude and longitude), we used EaserGeocoder, an open source geocoding software [15]. The geocoding process runs in-house, and therefore no sharing of patient data is needed. It was not possible to convert all patient addresses to geolocations, as some of them were either invalid or PO Box addresses instead of street addresses. These patient geolocations were added to the CT shapefile, then grouped and counted within a CT. OP and OUD rates per 100,000 persons were calculated for each CT. The SPARCS data also have patient-level demographic and other characteristics such as gender, age, race, and type of payment. We included 63,958 patients who had at least one OUD diagnosis between 2010 and 2016, and 36,004 patients with at least one OP diagnosis between 2012 and 2016.

There are 4919 total CTs in New York State according to ACS 2012-2016 5-year estimates. The 2016 voting data from Harvard Dataverse included data for 4900 (99.6%) CTs. After removing CTs with populations less than 100, 4836 (98.3%) CTs remained. These CTs were then used in the spatial mappings, of which 63 CTs had missing education data, 129 had missing income data, 61 had missing marital/race/gender data, and 69 had missing disability data. Excluding these CTs with missing values left 4777 (97.1%) CTs remaining for CT characteristic analyses.

### Analysis

The analyses were divided into 2 parts, one at the patient level using the SPARCS dataset and the other at the CT level while combining the CT dataset and the SPARCS dataset. First, descriptive statistics of patient-level characteristics were calculated for OUD and all patients in the SPARCS dataset. A logistic regression model was used to determine the associations between patient-level characteristics (eg, sex, age group, race, and payment type) with OUD. Odds ratios and their 95% confidence intervals were estimated based on the logistic regression. Second, maps for crude rates of opioid overdose normalized by population for OP and OUD and maps for 2016 Republican presidential vote rates were generated for CTs with ArcGIS. The OUD rates were heavily positively skewed due to the high resolution of the geography level and low counts of opioid use for each CT. Spearman rank order correlations were calculated to evaluate the association between OP/OUD and presidential election voting rates. The averages for CT-level demographics and socioeconomic factors were calculated and compared between the CTs with OUD rates in the lowest (1% to 25%) quartile and CTs with OUD rates in the highest (76% to 100%) quartile using *t* tests. To assess the extent to which the Republican presidential vote association with OUD is explained by CT-level characteristics, 3 regression models were built with the OUD rate as the dependent variable. Model 1 included only the percentage of voting for the Republican presidential candidate. Model 2 adjusted for CT demographic and socioeconomic features, and model 3 additionally aggregated medical factors and median age. Multicollinearity among covariates was evaluated using variation inflation factor. The standardized regression coefficients and partial *R*^2^ were reported. Statistical analyses were performed using R version 3.6.1 (R Foundation for Statistical Computing) and Python 3.7 (Python Software Foundation).

## Results

The patient-level characteristics of OUD and all patients in the SPARCS dataset are shown in Table 1. All patient-level characteristics are significantly associated with OUD. A male patient is 1.735 times more likely to have OUD than a female. The young adult (aged 18 to 24 years) age group is the most active in nonmedical opioid use, so this serves to be a good reference for odds ratio [16]. Compared with the young adult age group, all other age groups are less likely to have OUD (all OR <1.00, *P*<.001).

**Table 1:** Characteristics of Statewide Planning and Research Cooperative System database patients associated with opioid use disorders in New York State, 2010-2016.

Characteristics		All SPARCS^a^ (in and out) patients 2010-2016 (N=210,935,831), n (%)	OUD^b^ patients 2010-2016 (n=63,958), n (%)	Odds ratio (95% CI)	*P* value
**Sex**					
	Female	119,850,432 (56.18)	27,590 (43.13)	1 (Ref)	—^c^
	Male	91,076,257 (43.18)	36,366 (56.86)	1.73 (1.71-1.76)	<.001
**Age in years**					
	<18	26,893,357 (12.75)	1713 (2.68)	0.12 (0.11-0.12)	<.001
	18-24	17,276,865 (8.19)	9435 (14.75)	1 (Ref)	—
	25-39	38,832,961 (18.41)	19,402 (30.34)	0.92 (0.89-0.94)	<.001
	40-64	74,991,993 (35.55)	24,078 (37.65)	0.59 (0.58-0.60)	<.001
	≥65	46,078,300 (21.84)	9330 (14.59)	0.37 (0.36-0.38)	<.001
**Race**					
	White, non-Hispanic	90,255,132 (42.79)	42,764 (66.86)	1 (Ref)	—
	Black, non-Hispanic	41,978,134 (19.90)	6881 (10.76)	0.35 (0.34-0.36)	<.001
	Hispanic	50,616,600 (24.00)	9176 (14.35)	0.38 (0.37-0.39)	<.001
	Other	26,489,568 (12.56)	5097 (7.97)	0.41 (0.39-0.42)	<.001
**Payment type**					
	Insurance company	43,520,092 (20.63)	14,378 (22.48)	1.91 (1.87-1.95)	<.001
	Medicare	28,390,633 (13.46)	14,564 (22.77)	1.26 (1.23-1.29)	<.001
	Medicaid	17,920,248 (8.50)	6390 (9.99)	1.34 (1.31-1.38)	<.001
	Self-pay	28,652,602 (13.58)	8810 (13.77)	1.16 (1.13-1.19)	<.001
	Other	43,520,092 (20.63)	14,378 (22.48)	1.91 (1.87-1.95)	<.001

^a^SPARCS: Statewide Planning and Research Cooperative System.

^b^OUD: opioid use disorder.

^c^Not applicable.

**Figure 1:**
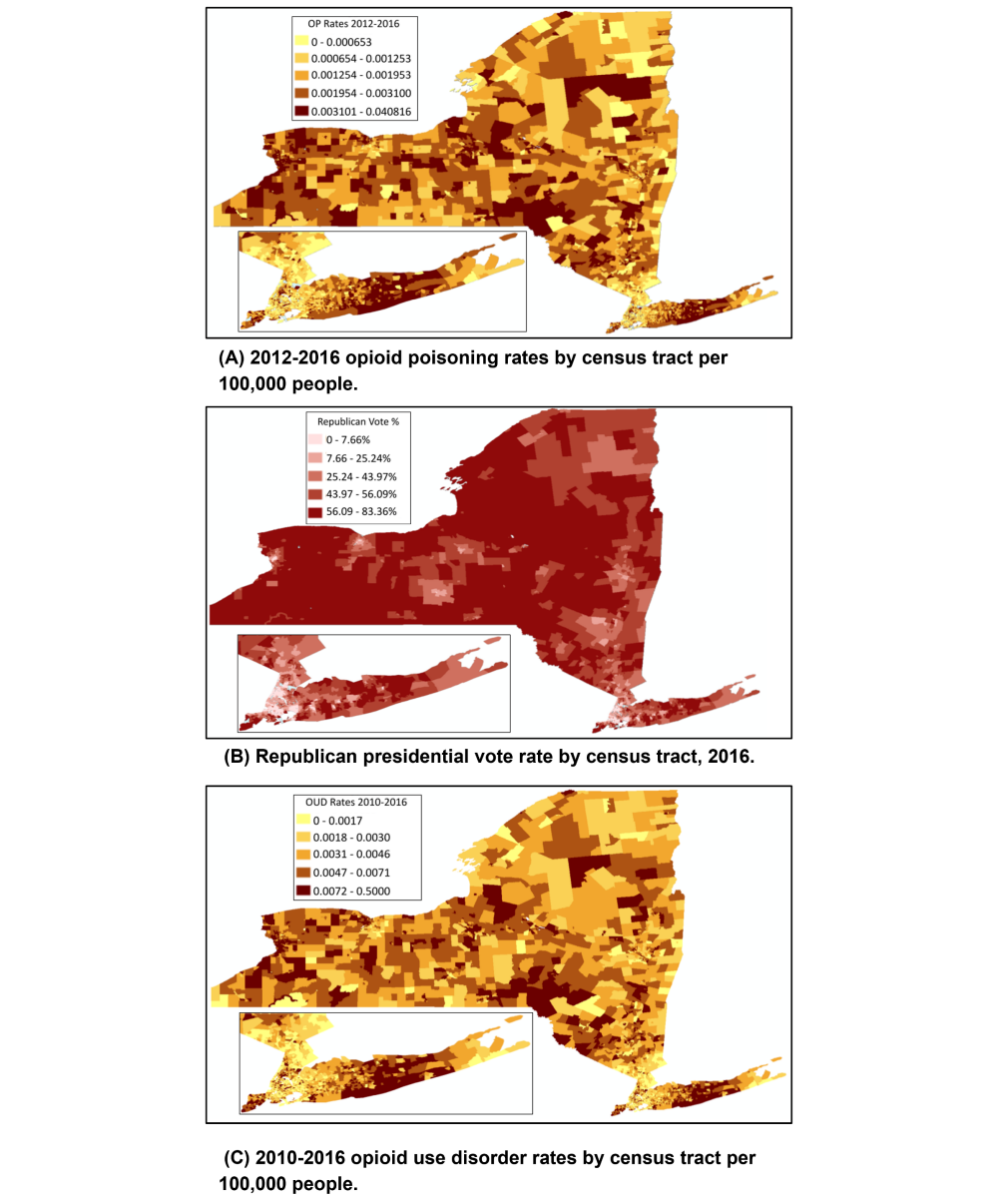
Opioid use and Republican presidential vote 2016 in New York State at census tract level: (A) 2012-2016 opioid poisoning rates by census tract per 100,000 people, (B) Republican presidential vote rate by census tract, 2016, (C) 2010-2016 opioid use disorder rates by census tract per 100,000 people.

Figure 1 illustrates opioid use and Republican voting in 4836 of 4919 CTs in New York State. Each map has color ranges ordered by quintiles at the CT level. The first map (A) shows OP rates 2012-2016 with a closer look into New York City and Long Island, which have rates among the highest in the state [13]. About 1 in 5 CTs had more than 310 OP diagnoses per 100,000 persons, while a similar proportion had fewer than 65 diagnoses per 100,000 persons. The areas of higher OP diagnoses were Suffolk County on eastern Long Island, Erie County in western New York, Oneida/Onondaga Counties in central New York, and Delaware/Broome Counties in the Southern Tier. Metro areas varied in OP rates. The second map (B) portrays the percentage of the presidential vote for the Republican candidate for each CT. Note that large urban areas had, for the most part, lower support for the Republican candidate. Several areas shared similar patterns to the OP rates shown in map A: primarily, CTs in western New York, central New York, and Suffolk County. The third map (C) shows OUD rates 2010-2016 at the CT level. The results are similar to map A. The spearman correlation between maps A and B was 0.38 (*P*<.001), between maps B and C was 0.38 (*P*<.001), and between maps A and C was 0.86 (*P*<.001).

Next, we examined the CT-level characteristics between the Republican presidential vote and opioid use. In Table 2, we tested the differences in the average of various socioeconomic and demographic features at the CT level between the low- and high-OUD CTs. CTs were ranked by OUD rates, and the lowest and highest quartiles were used for comparison. The Republican presidential vote demonstrated the highest differences between high- and low-OUD rate CTs, with the former voting at an average rate of 42.86% (SE 0.56%, *P*<.001) for the Republican candidate, more than twice the average rate of 20.85% (SE 0.55%) for lower OUD rate CTs. Other characteristics with relatively large interquartile differences include percentage of population with disabilities, percentage of white population, and percentage of households in urban areas.

**Table 2:** Characteristics compared between census tracts with lower opioid use versus census tracts with higher opioid use in New York State, 2010-2016.

Characteristics	Total (n=4777), mean (SE)	Lower OUD^a^ rate tracts (n=1193), mean (SE)	Higher OUD rate tracts (n=1194), mean (SE)	Mean difference higher-lower (95% CI)	*P* value
Disability %	11.59 (0.07)	9.31 (0.11)	14.45 (0.17)	5.14 (4.74 to 5.54)	<.001
Republican vote %	33.66 (0.32)	20.85 (0.55)	42.86 (0.56)	22.01 (20.47 to 23.55)	<.001
Marriage %	44.39 (0.19)	43.45 (0.38)	41.79 (0.38)	–1.66 (–2.71 to –0.61)	.002
Urban %	87.45 (0.43)	95.61 (0.55)	86.09 (0.90)	–9.52 (–11.59 to –7.45)	<.001
Male %	48.52 (0.06)	48.14 (0.13)	48.74 (0.12)	0.60 (0.25 to 0.95)	.0007
White %	63.69 (0.46)	47.90 (0.92)	74.71 (0.77)	26.81 (24.46 to 29.16)	<.001
Unemployment %	7.96 (0.07)	7.95 (0.14)	8.67 (0.16)	0.72 (0.30 to 1.14)	.0007
Medicare eligible %	10.47 (0.07)	11.93 (0.16)	9.40 (0.12)	–2.53 (–2.92 to –2.14)	<.001
Medicaid eligible %	12.92 (0.07)	13.79 (0.16)	12.15 (0.13)	–1.64 (–2.04 to –1.24)	<.001
High school diploma %	27.27 (0.14)	24.17 (0.30)	31.60 (0.23)	7.43 (6.68 to 8.20)	<.001
Median age	39.15 (0.10)	37.20 (0.20)	39.87 (0.20)	2.67 (2.12 to 3.22)	<.001
Median income ($)	65,913.31 (467.12)	69,461.49 (1003.40)	55,756.10 (790.90)	–13,705.39 (–16,210.84 to –11,199.94)	<.001

^a^OUD: opioid use disorder.

Finally, we analyzed the extent to which the Republican presidential vote explains the variation of OUD rates with adjustment for CT-level characteristics. Table 3 shows 3 multiple linear regression models with adjustment for CT characteristics. Model 1 only includes the percentage of Republican vote, which shows a positive relationship and explains 5% of the county-level variation in opioid use. Model 2 accounts for several CT characteristics in addition to the Republican vote and explains 24% of the variation in OUD rates. The percentage of Republican vote explains 3% of the variation in OUD rates. Model 3 includes all of the characteristics in Table 2, adding health care–related factors (Medicare eligibility and disability) as well as median age. Medicaid was not included because it had a high collinearity with Medicare (variation inflation factor = 7.5, correlation = 0.88). The model explains 30% of the variation in OUD rates, and the percentage of Republican votes explains 1% of the variation in OUD rates. From models 2 and 3, the most prominent variables that explain the variation in CT OUD rates are disability rates, percentage of Republican vote, and marriage rates.

**Table 3:** Socioeconomic and demographic factors associating the Republican vote with opioid use disorder rates per 100,000 people, 2010-2016.

Characteristics			*R* ^2^	Partial *R*^2^		Standardized regression coefficient		Standard error		*P* value
**Model 1**			.05	—^a^		—		—		—
	Intercept	—		—	<.001		.01		1	
	Republican vote %	—		.05	.23		.01		<.001	
**Model 2**			.24	—		—		—		—
	Intercept	—		—	<.001		.01		1	
	Marriage %	—		.07	–.40		.02		<.001	
	Republican vote %	—		.03	.32		.03		<.001	
	White %	—		.03	.28		.03		<.001	
	Urban household %	—		.02	.14		.01		<.001	
	High school diploma %	—		.01	.13		.02		<.001	
	Unemployment %	—		.01	.12		.02		<.001	
	Male %	—		.004	.06		.01		<.001	
	Median income, per $1000	—		.002	–.02		.02		.35	
**Model 3**			.30	—		—		—		—
	Intercept	—		—	<.001		.01		1	
	Marriage %	—		.04	–.32		.02		<.001	
	Disability %	—		.05	.27		.02		<.001	
	Republican vote %	—		.01	.24		.03		<.001	
	White %	—		.02	.22		.02		<.001	
	Urban %	—		.03	.17		.01		<.001	
	High school diploma %	—		.01	.13		.02		<.001	
	Male %	—		.01	.09		.01		<.001	
	Medicare eligible	—		.006	–.07		.01		<.001	
	Unemployment %	—		.003	.06		.02		<.001	
	Median income $	—		.002	.06		.02		.001	
	Median age	—		.0005	.03		.02		.12	

^a^Not applicable.

## Discussion

### Principal Findings

The demographic findings for OUD in New York State were generally consistent with recently published epidemiology of the US opioid epidemic in that young adult white males are overrepresented [16].

We have explored the specific geographic relationships between opioids, voting patterns, and demographic features like disability and unemployment. Disability may be the easiest factor to explain. In the United States, the largest proportion of years lived with a disability is attributable to chronic noncancer pain, and globally, musculoskeletal (ie, back and neck) pain is the third leading cause of disability-adjusted life-years [17]. As chronic pain is well described as the most common source of chronic disability in the United States, and opioid treatment is also well described as increasing the odds ratio for the development of OUD, especially with chronic exposure [18,19], it is reasonable to expect that the odds ratio for OUD is increased in patients with chronic disabling conditions. Additionally, OUD related to use of prescription pain medications is highly disabling, which offers another linkage to our finding [20].

Next, the small but significant contribution of differences in marriage status is also meaningful in the context of social and economic changes that have paralleled and likely contributed to the arc of the opioid epidemic over the past 25 years [21]. In our analysis, the interquartile differences demonstrated a small but significant negative correlation between marriage percentage and OUD. Monnat and Brown [22] describe “landscapes of despair”—the small cities and rural areas where over several decades social and family conditions have been deteriorating as economic distress (eg, job loss due to manufacturing and natural resource industry decline) has been mounting. They found, consistent with our findings, the highest percentage of 2016 Republican voting over the 2012 baseline in the top quartile of counties with the lowest well-being, which included higher separation and divorce rates as compared with the quartile of counties with the highest well-being [22]. These locales are also where the 2016 Republican candidate overperformed compared with Republican voting patterns in the 2012 election: counties with the highest rate of deaths of despair (ie, those with the highest drug, alcohol, and suicide mortality rates attributable in large measure to economic distress and a large working class) [23]. The interquartile comparisons between high- and low-OUD rate CTs in Table 2 resonate with these landscapes of despair in that in addition to the large difference (>100%) in Republican vote, they generally demonstrate face validity in the valence of the correlations in the high-OUD tracts: higher percentage White, more disability, unemployment, high school diploma as terminal degree, and male gender with less marriage, urbanicity, and median income.

Understanding these landscapes of despair is crucial because opioids are an anodyne to both physical and emotional pain. Whereas life expectancy continues to rise in wealthy market economies, recent studies reveal a grim picture of increasing morbidity and all-cause mortality of middle-aged white non-Hispanic US men and women since 1999, mostly due to drug and alcohol poisonings, suicide, and alcohol-related liver diseases, especially among those with high school education or less [24,25]. In addition, compared with college-educated people, since 1950, those without a bachelor’s degree have a higher prevalence of pain at each age, a prevalence that is increasing with each successive birth cohort [26]. Among validated voters in 2016, the Republican candidate won by more than 2 to 1 (64% to 28%) among white voters who had not completed college (44% of all voters), which aligns with the demographics of OUD [27]. These facts may also help explain the relationship between opioids and voting patterns. A political candidate might have appealed to the residents of these landscapes by resonating with their emotional and physical needs, their sense of lost status, opportunity, and agency, and presenting themself as a kind of anodyne by promising to uplift them economically and/or sociopolitically [28].

We have shown that Republican voting percentage is independently associated with OUD in model 1 and remains significant in model 2 and 3 with adjustment for other covariates. This is sufficient to show Republican voting percentage is an important associated factor of OUD. Although a causal relationship cannot be inferred, our model clusters lower odds of having a marital partner, increased disability, voting Republican, and high school diploma as a terminal degree with risk for OUD, as well as being male, white, urban, and unemployed. Our findings highlight the relationship between OUD and factors related to despair, suggesting that socioeconomic growth may be necessary to successfully fight the opioid epidemic, in addition to traditional interventions like improved access to OUD treatment. Disability, unemployment, and nonmarried status do not have to cause despair but are likely to do so in communities that lack a safety net, both economically and socially. Understanding and responding to the needs of these “landscapes of despair” may be key to reversing the opioid epidemic and may also affect the political direction of the United States.

### Limitations

Our study has a few limitations regarding the method and underlying assumptions about the population. It is important to note that the population base containing the sample that voted Republican in 2016 is not the same as the population base data we used to determine OUD but rather they were generalized and configured to the CT level. In addition, for the purposes of constructing our statistical analyses, we assumed in these populations that socioeconomic and demographic factors affect OUD rates. All associations in our study were found to be mild. This could partially be due to the retrospective study design and inaccuracy in the aggregated census data. In the future, a well-designed prospective study may reveal more accurately the influence of socioeconomic and demographic factors on OUD. Last, in order to converge the datasets appropriately, we assumed that the population in which we drew data to determine OUD was also alive and voting in the 2016 election.

### Conclusions

The association between the 2016 Republican presidential vote and OUD highlights the demographic, geographic, and socioeconomic characteristics that underpin both features. Studying opioid use at a finer grain geospatial level provides a unique opportunity for a more precise understanding of the opioid epidemic at large scale.

## Appendix

Multimedia Appendix 1 Illustration of data conversion from precinct level to census tract level. The blue-outlined polygon is a census tract and the black-outlined polygons are precincts. To convert precinct level election data to the census tract level, precinct areas within each census tract were calculated (table in figure). The linear combination of voting counts and area percentage provides an estimate of the vote counts at the census tract level.

Multimedia Appendix 2 International Classification of Diseases (ICD)-9 and ICD-10 diagnosis codes related to opioid poisoning.

Multimedia Appendix 3 International Classification of Diseases (ICD)-9 and ICD-10 diagnosis codes related to opioid use disorder.

## Abbreviations

ACS: American Community Survey
CT: census tract
ICD: International Classification of Diseases
OUD: opioid use disorder
OP: opioid poisoning
SPARCS: Statewide Planning and Research Cooperative System
