# Supplementary figures and images for: Association of Opioid Use Disorder With 2016 Presidential Voting Patterns: Cross-sectional Study in New York State at Census Tract Level
Source: JMIR Public Health Surveill. 2021 Apr 21;7(4):e23426. doi: 10.2196/23426 (PMC8100884; doi:10.2196/23426)

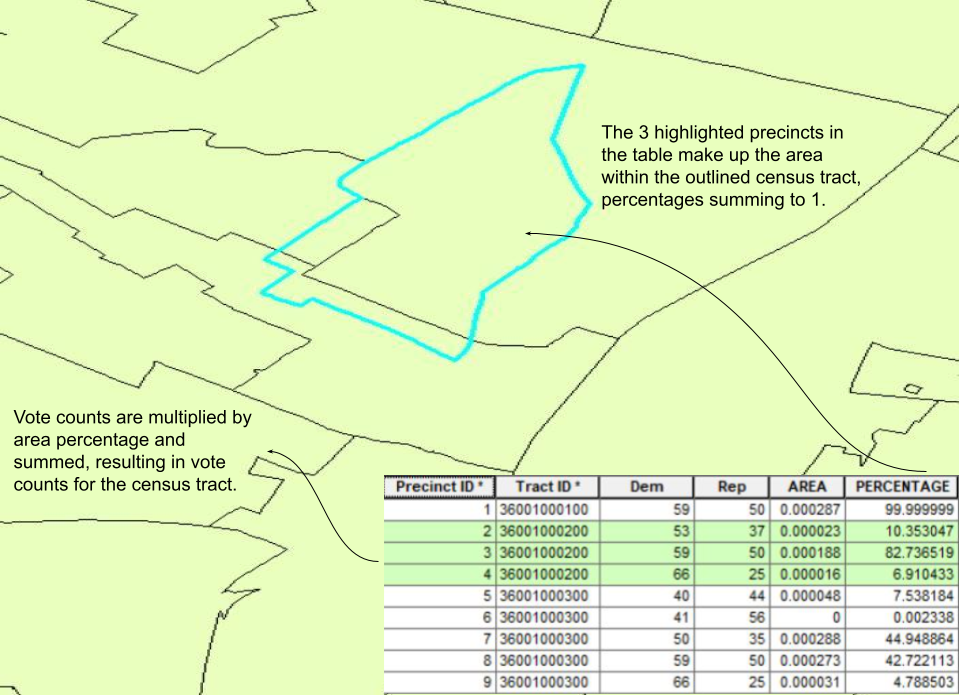

Supplement: Multimedia Appendix 1 [file publichealth_v7i4e23426_app1.png]
