# Supplementary material for: Association of Opioid Use Disorder With 2016 Presidential Voting Patterns: Cross-sectional Study in New York State at Census Tract Level
Source: JMIR Public Health Surveill. 2021 Apr 21;7(4):e23426. doi: 10.2196/23426 (PMC8100884; doi:10.2196/23426)
Supplement: Multimedia Appendix 2 [file publichealth_v7i4e23426_app2.docx]

| Years | ICD Code | Version | Description |
| --- | --- | --- | --- |
| 2012-2015 | 9650 | 9 | Poisoning; Opiates and Related Narcotics |
| 2012-2015 | 96500 | 9 | Poisoning; Opium (alkaloids), unspecified |
| 2012-2015 | 96501 | 9 | Poisoning; Heroin |
| 2012-2015 | 96502 | 9 | Poisoning; Methadone |
| 2012-2015 | 96509 | 9 | Poisoning; Other |
| 2012-2015 | E8500 | 9 | Accidental Poisoning; Heroin |
| 2012-2015 | E8501 | 9 | Accidental Poisoning; Methadone |
| 2010-2015 | E8502 | 9 | Accidental Poisoning; Other Opiates and Related Narcotics |
| 2015-2016 | T400X1A | 10 | Poisoning by opium, accidental (unintentional initial encounter) |
| 2015-2016 | T400X1S | 10 | Poisoning by opium, accidental (unintentional), sequela |
| 2015-2016 | T400X2A | 10 | Poisoning by opium, intentional self-harm, initial encounter |
| 2015-2016 | T400X2S | 10 | Poisoning by opium, intentional self-harm, sequela |
| 2015-2016 | T400X3A | 10 | Poisoning by opium, assault, initial encounter |
| 2015-2016 | T400X3S | 10 | Poisoning by opium, assault, sequela |
| 2015-2016 | T400X4A | 10 | Poisoning by opium, undetermined, initial encounter |
| 2015-2016 | T400X4S | 10 | Poisoning by opium, undetermined, sequela |
| 2015-2016 | T400X5A | 10 | Adverse effect of opium, initial encounter |
| 2015-2016 | T400X5S | 10 | Adverse effect of opium, sequela |
| 2015-2016 | T401X1A | 10 | Poisoning by heroin, accidental (unintentional), initial encounter |
| 2015-2016 | T401X1S | 10 | Poisoning by heroin, accidental (unintentional), sequela |
| 2015-2016 | T401X2A | 10 | Poisoning by heroin, intentional self-harm, initial encounter |
| 2015-2016 | T401X2S | 10 | Poisoning by heroin, intentional self-harm, sequela |
| 2015-2016 | T401X3A | 10 | Poisoning by heroin, assault, initial encounter |
| 2015-2016 | T401X3S | 10 | Poisoning by heroin, assault, sequela |
| 2015-2016 | T401X4A | 10 | Poisoning by heroin, undetermined, initial encounter |
| 2015-2016 | T401X4S | 10 | Poisoning by heroin, undetermined, sequela |
| 2015-2016 | T402X1A | 10 | Poisoning by other opioids, accidental (unintentional), initial encounter |
| 2015-2016 | T402X1S | 10 | Poisoning by other opioids, accidental, sequela |
| 2015-2016 | T402X2A | 10 | Poisoning by other opioids, intentional self-harm, initial encounter |
| 2015-2016 | T402X2S | 10 | Poisoning by other opioids, intentional self-harm, sequela |
| 2015-2016 | T402X3A | 10 | Poisoning by other opioids, assault, initial encounter |
| 2015-2016 | T402X3S | 10 | Poisoning by other opioids, assault, sequela |
| 2015-2016 | T402X4A | 10 | Poisoning by other opioids, undetermined, initial encounter |
| 2015-2016 | T402X4S | 10 | Poisoning by other opioids, undetermined, sequela |
| 2015-2016 | T402X5A | 10 | Adverse effect of other opioids, initial encounter |
| 2015-2016 | T402X5S | 10 | Adverse effect of other opioids, sequela |
| 2015-2016 | T403X1A | 10 | Poisoning by methadone, accidental (unintentional), initial encounter |
| 2015-2016 | T403X1S | 10 | Poisoning by methadone, accidental (unintentional), sequela |
| 2015-2016 | T403X2A | 10 | Poisoning by methadone, intentional self-harm, initial encounter |
| 2015-2016 | T403X2S | 10 | Poisoning by methadone, intentional self-harm, sequela |
| 2015-2016 | T403X3A | 10 | Poisoning by methadone, assault, initial encounter |
| 2015-2016 | T403X3S | 10 | Poisoning by methadone, assault, sequela |
| 2015-2016 | T403X4A | 10 | Poisoning by methadone, undetermined, initial encounter |
| 2015-2016 | T403X4S | 10 | Poisoning by methadone, undetermined, sequela |
| 2015-2016 | T403X5A | 10 | Adverse effect of methadone, initial encounter |
| 2015-2016 | T403X5S | 10 | Adverse effect of methadone, sequela |
| 2015-2016 | T404X1A | 10 | Poisoning by other synthetic narcotics, accidental, initial encounter |
| 2015-2016 | T404X1S | 10 | Poisoning by other synthetic narcotics, accidental, sequela |
| 2015-2016 | T404X2A | 10 | Poisoning by other synthetic narcotics, self-harm, initial encounter |
| 2015-2016 | T404X2S | 10 | Poisoning by other synthetic narcotics, self-harm, sequela |
| 2015-2016 | T404X3A | 10 | Poisoning by other synthetic narcotics, assault, initial encounter |
| 2015-2016 | T404X3S | 10 | Poisoning by other synthetic narcotics, assault, sequela |
| 2015-2016 | T404X4A | 10 | Poisoning by other synthetic narcotics, undetermined, initial encounter |
| 2015-2016 | T404X4S | 10 | Poisoning by other synthetic narcotics, undetermined, sequela |
| 2015-2016 | T404X5A | 10 | Adverse effect of other synthetic narcotics, initial encounter |
| 2015-2016 | T404X5S | 10 | Adverse effect of other synthetic narcotics, sequela |
