# Supplementary material for: Association of Opioid Use Disorder With 2016 Presidential Voting Patterns: Cross-sectional Study in New York State at Census Tract Level
Source: JMIR Public Health Surveill. 2021 Apr 21;7(4):e23426. doi: 10.2196/23426 (PMC8100884; doi:10.2196/23426)
Supplement: Multimedia Appendix 3 [file publichealth_v7i4e23426_app3.docx]

| **Years** | **Version** | **ICD Code** | **Description** |
| --- | --- | --- | --- |
| 2012-2015 | 9 | 30400 | Opioid dependence-unspecified |
| 2012-2015 | 9 | 30401 | Opioid dependence-continuous |
| 2012-2015 | 9 | 30402 | Opioid dependence-episode |
| 2012-2015 | 9 | 30403 | Opioid type dependence in remission |
| 2012-2015 | 9 | 30470 | Opioid/other dep-unspecified |
| 2012-2015 | 9 | 30471 | Opioid/other dep-continuous |
| 2012-2015 | 9 | 30472 | Opioid/other dep-episode |
| 2012-2015 | 9 | 30473 | Opioid w/other drug dependence in remission |
| 2012-2015 | 9 | 30550 | Opioid abuse-unspecified |
| 2012-2015 | 9 | 30551 | Opioid abuse-continuous |
| 2012-2015 | 9 | 30552 | Opioid abuse-episodic |
| 2012-2015 | 9 | 30553 | Opioid abuse in remission |
| 2012-2015 | 9 | 96500 | Poisoning by opium (alkaloids), unspecified |
| 2012-2015 | 9 | 96501 | Poisoning by heroin |
| 2012-2015 | 9 | 96502 | Poisoning by methadone |
| 2012-2015 | 9 | 96509 | Poisoning by other opiates |
| 2012-2015 | 9 | E8500 | Accidental poisoning by heroin |
| 2012-2015 | 9 | E8501 | Accidental poisoning by methadone |
| 2012-2015 | 9 | E8502 | Accidental poisoning by other opiates and related narcotics |
| 2012-2015 | 9 | E9800 | Undetermined cause poisoning by opiates |
| 2015-2016 | 10 | F111 | Opioid abuse |
| 2015-2016 | 10 | F1110 | Opioid abuse uncomplicated |
| 2015-2016 | 10 | F1112 | Opioid abuse with intoxication |
| 2015-2016 | 10 | F11120 | Opioid abuse with intoxication uncomplicated |
| 2015-2016 | 10 | F11121 | Opioid abuse with intoxication delirium |
| 2015-2016 | 10 | F11122 | Opioid abuse w/intoxication w/perceptual disturb |
| 2015-2016 | 10 | F11129 | Opioid abuse with intoxication unspecified |
| 2015-2016 | 10 | F1114 | Opioid abuse with opioid-induced mood disorder |
| 2015-2016 | 10 | F1115 | Opioid abuse with opioid-induced psychotic disorder |
| 2015-2016 | 10 | F11150 | Opioid abuse w/induced psychosis d/o w/delusions |
| 2015-2016 | 10 | F11151 | Opioid abuse w/induced psychosis d/o w/hallucinations |
| 2015-2016 | 10 | F11159 | Opioid abuse w/opioid-induced psychosis d/o unspecified |
| 2015-2016 | 10 | F1118 | Opioid abuse with other opioid-induced disorder |
| 2015-2016 | 10 | F11181 | Opioid abuse w/opioid-induced sexual dysfunction |
| 2015-2016 | 10 | F11182 | Opioid abuse with opioid-induced sleep disorder |
| 2015-2016 | 10 | F11188 | Opioid abuse with other opioid-induced disorder |
| 2015-2016 | 10 | F1119 | Opioid abuse w/unspecified opioid-induced disorder |
| 2015-2016 | 10 | F112 | Opioid dependence |
| 2015-2016 | 10 | F1120 | Opioid dependence, uncomplicated |
| 2015-2016 | 10 | F1121 | Opioid dependence, in remission |
| 2015-2016 | 10 | F11220 | Opioid dependence with intoxication, uncomplicated |
| 2015-2016 | 10 | F11221 | Opioid dependence with intoxication delirium |
| 2015-2016 | 10 | F1122 | Opioid dependence with intoxication |
| 2015-2016 | 10 | F11222 | Opioid dependence with intoxication with perceptual disturbance |
| 2015-2016 | 10 | F11229 | Opioid dependence with intoxication, unspecified |
| 2015-2016 | 10 | F1123 | Opioid dependence with withdrawal |
| 2015-2016 | 10 | F1124 | Opioid dependence with opioid-induced mood disorder |
| 2015-2016 | 10 | F1125 | Opioid dependence with opioid-induced psychotic disorder |
| 2015-2016 | 10 | F11250 | Opioid dependence with opioid-induced psychotic disorder with delusions |
| 2015-2016 | 10 | F11251 | Opioid dependence with opioid-induced psychotic disorder with hallucinations |
| 2015-2016 | 10 | F11259 | Opioid dependence with opioid-induced psychotic disorder, unspecified |
| 2015-2016 | 10 | F1128 | Opioid dependence with other opioid-induced disorder |
| 2015-2016 | 10 | F11281 | Opioid dependence with opioid-induced sexual dysfunction |
| 2015-2016 | 10 | F11282 | Opioid dependence with opioid-induced sleep disorder |
| 2015-2016 | 10 | F11288 | Opioid dependence with other opioid-induced disorder |
| 2015-2016 | 10 | F1129 | Opioid dependence with unspecified opioid-induced disorder |
| 2015-2016 | 10 | F1190 | Opioid use, unspecified, uncomplicated |
| 2015-2016 | 10 | F11920 | Opioid use, unspecified with intoxication, uncomplicated |
| 2015-2016 | 10 | F11921 | Opioid use, unspecified with intoxication delirium |
| 2015-2016 | 10 | F11922 | Opioid use, unspecified with intoxication with perceptual disturbance |
| 2015-2016 | 10 | F11929 | Opioid use, unspecified with intoxication, unspecified |
| 2015-2016 | 10 | F1193 | Opioid use, unspecified with withdrawal |
| 2015-2016 | 10 | F1194 | Opioid use, unspecified with opioid-induced mood disorder |
| 2015-2016 | 10 | F11950 | Opioid use, unspecified with opioid-induced psychotic disorder with delusions |
| 2015-2016 | 10 | F11951 | Opioid use, unspecified with opioid-induced psychotic disorder with hallucinations |
| 2015-2016 | 10 | F11959 | Opioid use, unspecified with opioid-induced psychotic disorder, unspecified |
| 2015-2016 | 10 | F11981 | Opioid use, unspecified with opioid-induced sexual dysfunction |
| 2015-2016 | 10 | F11982 | Opioid use, unspecified with opioid-induced sleep disorder |
| 2015-2016 | 10 | F11988 | Opioid use, unspecified with other opioid-induced disorder |
| 2015-2016 | 10 | F1199 | Opioid use, unspecified with unspecified opioid-induced disorder |
| 2015-2016 | 10 | T400X1A | Poisoning by opium, accidental (unintentional), initial encounter |
| 2015-2016 | 10 | T400X2A | Poisoning by opium, intentional self-harm, initial encounter |
| 2015-2016 | 10 | T400X4A | Poisoning by opium, undetermined, initial encounter |
| 2015-2016 | 10 | T401X1A | Poisoning by heroin, accidental (unintentional), initial encounter |
| 2015-2016 | 10 | T401X2A | Poisoning by heroin, intentional self-harm, initial encounter |
| 2015-2016 | 10 | T401X4A | Poisoning by heroin, undetermined, initial encounter |
| 2015-2016 | 10 | T402X1A | Poisoning by other opioids, accidental (unintentional), initial encounter |
| 2015-2016 | 10 | T402X2A | Poisoning by other opioids, intentional self-harm, initial encounter |
| 2015-2016 | 10 | T402X4A | Poisoning by other opioids, undetermined, initial encounter |
| 2015-2016 | 10 | T403X1A | Poisoning by methadone, accidental (unintentional), initial encounter |
| 2015-2016 | 10 | T403X2A | Poisoning by methadone, intentional self-harm, initial encounter |
| 2015-2016 | 10 | T403X4A | Poisoning by methadone, undetermined, initial encounter |
